# Safety, Efficacy, and Biological Data of T-Cell–Enabling Oncolytic Adenovirus TILT-123 in Advanced Solid Cancers from the TUNIMO Monotherapy Phase I Trial

**DOI:** 10.1158/1078-0432.CCR-23-3874

**Published:** 2024-03-27

**Authors:** Santeri A. Pakola, Katriina J. Peltola, James H.A. Clubb, Elise Jirovec, Lyna Haybout, Tatiana V. Kudling, Tuomo Alanko, Riitta Korpisaari, Susanna Juteau, Marjut Jaakkola, Jorma Sormunen, Jukka Kemppainen, Annabrita Hemmes, Teijo Pellinen, Mirte van der Heijden, Dafne C.A. Quixabeira, Claudia Kistler, Suvi Sorsa, Riikka Havunen, Joao M. Santos, Victor Cervera-Carrascon, Akseli Hemminki

**Affiliations:** 1 Cancer Gene Therapy Group, Translational Immunology Research Program, University of Helsinki, Helsinki, Finland.; 2 Comprehensive Cancer Center, Helsinki University Hospital, Helsinki, Finland.; 3 TILT Biotherapeutics Ltd., Helsinki, Finland.; 4 Docrates Cancer Center, Helsinki, Finland.; 5 Department of Pathology, University of Helsinki and Helsinki University Hospital, Helsinki, Finland.; 6 Digital Microscopy and Molecular Pathology Unit, Institute for Molecular Medicine Finland, Helsinki, Finland.

## Abstract

**Purpose:**

TILT-123 (igrelimogene litadenorepvec) is an oncolytic adenovirus armed with TNFa and IL2, designed to induce T-cell infiltration and cytotoxicity in solid tumors.

**Patients and Methods:**

TUNIMO (NCT04695327) was a single-arm, multicenter phase I dose-escalation trial designed to assess the safety of TILT-123 in advanced solid cancers refractory to standard therapy. Patients received intravenous and intratumoral TILT-123. The primary endpoint was safety by adverse events (AE), laboratory values, vital signs, and electrocardiograms. Secondary endpoints included tumor response, pharmacokinetics, and predictive biomarkers.

**Results:**

Twenty patients were enrolled, with a median age of 58 years. Most prevalent cancer types included sarcomas (35%), melanomas (15%) and ovarian cancers (15%). No dose-limiting toxicities were observed. The most frequent treatment-related AEs included fever (16.7%), chills (13.0%), and fatigue (9.3%). Ten patients were evaluable for response on day 78 with RECIST 1.1, iRECIST or PET-based evaluation. The disease control rate by PET was 6/10 (60% of evaluable patients) and 2/10 by RECIST 1.1 and iRECIST(20%of evaluable patients). Tumor size reductions occurred in both injected and non-injected lesions. TILT-123 was detected in injected and non-injected tumors, and virus was observed in blood after intravenous and intratumoral injections. Treatment resulted in reduction of lymphocytes in blood, with concurrent lymphocyte increases in tumors, findings compatible with trafficking.

**Conclusions:**

TILT-123 was safe and able to produce antitumor effects in local and distant lesions in heavily pre-treated patients. Good tolerability of TILT-123 facilitates combination studies, several of which are ongoing (NCT04217473, NCT05271318, NCT05222932, and NCT06125197).

*See related commentary by Silva-Pilipich and Smerdou, p. 3649*

**Translational Relevance**

TUNIMO describes the first results of TILT-123 (igrelimogene litadenorepvec), an oncolytic adenovirus encoding TNFa and IL2, in humans with advanced solid cancers. This phase I study showed good tolerability of TILT-123 therapy. TILT-123 was detected in blood and in both injected and non-injected tumors following intravenous and intratumoral dosing, demonstrating systemic delivery. Accordingly, antitumor effects were seen in both injected and non-injected tumors, with long-term survival of some patients. Correlative analyses showed that although neutralizing antibodies against the virus were generated in all patients, antibodies did not interfere with therapeutic effect. Serum proteomic analysis revealed upregulation of pro-inflammatory and chemotactic proteins after intravenous and intratumoral administration. This study demonstrates the safety of TILT-123 as a cancer therapy and lays the groundwork for future studies of TILT-123 in specific indications and with combination therapies.

## Introduction

Despite encouraging results in a proportion of patients, most solid cancers remain unresponsive to immunotherapy. Reasons for immune checkpoint inhibitor (ICI) immunotherapy failure in solid tumors are multifaceted, but two key recognized mechanisms are poor trafficking of effector immune cells to tumors and anergic state of T cells found in tumors (1). To resolve these challenges, an oncolytic adenovirus TILT-123 (igrelimogene litadenorepvec) was designed (2–4). TILT-123 is a serotype 5 based adenovirus featuring a serotype 3 fiber knob to facilitate systemic delivery and more efficient entry into cancer cells (5, 6). Cancer-specific replication is achieved using a dual selectivity device incorporating a 24 kb deletion in the adenovirus E1A gene together with a E2F promoter inserted upstream of the E1 region (2). These modifications allow the virus to replicate preferentially in p16/Rb pathway defective cells, including most advanced solid tumors, as this pathway is universally abnormal in cancer (7–10).

Oncolytic viruses are an emerging approach for cancer therapy. Although some products such as Imlygic (oncolytic HSV-1 approved for use in melanoma), Oncorine (oncolytic adenovirus approved in nasopharyngeal carcinoma), and Delytact (oncolytic HSV-1 approved in glioma), have already received regulatory approval, most phase 1 trials have suggested insufficient single-agent activity and few agents have proceeded to phase 2. In laboratory investigations featuring immune defective models, the emphasis has been on the oncolytic potential of agents. In contrast, when used in humans, it became apparent that an important consequence of oncolysis is induction of a T-cell dominant immune reaction against the tumor (11, 12). This generated the rationale for making an oncolytic adenovirus optimized for T-cell stimulation (7, 13, 14).

TILT-123 encodes two transgenes: TNFa and IL2, which were chosen following a detailed laboratory investigation aiming at identifying the optimal molecules for T-cell recruitment and activation (3, 4, 15). TNFa was chosen for its ability to achieve immune cell trafficking to the tumors, but it also can cause direct cancer cell death. IL2 was chosen for its ability to boost T-cell proliferation and support cytotoxic effector functions within tumors. In addition to production of the transgenes, TILT-123 lyses cancer cells as a result of the viral replication cycle, facilitating tumor antigen spread, pathogen/damage-associated molecular pattern spread and boosting antitumor immunity (4, 16, 17).

In the present article, we describe the first full trial results of TILT123 in humans. TUNIMO (NCT04695327) was a multicenter phase I dose-escalation trial of TILT-123 monotherapy assessing the safety and anti-tumor efficacy of TILT-123 in solid tumors refractory to standard therapy.

## Patients and Methods

### Patients

Between February 18, 2021, and July 13, 2023, 20 patients were enrolled in the trial. Inclusion criteria included cancer disease where standard therapy had failed or did not exist; at least one tumor available for intratumoral injection; adequate hematological [hemoglobin >100 g/L, white blood cells (WBC) >3.0 E9/L, platelets >75,000/mm^3^], hepatic [aspartate aminotransferase (AST), alanine aminotransferase (ALT) < 3 upper limit of normal (ULN) and bilirubin < 1.5 ULN], and renal function [glomerular filtration rate (GFR) >60 mL/min]; World Health Organization/Eastern Cooperative Oncology Group (WHO/ECOG) performance score of 0–1 at screening; and life expectancy longer than 3 months. Exclusion criteria included use of immunosuppressive medications (corticosteroids or drugs used in autoimmune diseases), treatment with anticancer therapy within 30 days, history of severe liver disease or coagulation disorder, uncontrolled cardiac or vascular disease, or previous therapy with oncolytic virus.

All patients gave written informed consent. The trial protocol and ethics were reviewed by the Finnish Medical Agency and the Helsinki University Hospital Ethics board (approval 49/2020 and statement HUS/1804/2020).

### Procedures

#### Production of TILT-123

TILT-123 was manufactured according to Good Manufacturing Practices in A549 cells. Before administration, TILT-123 was resuspended in 0.9% saline and administered in 1.0 to 5.0 mL volume for intratumoral injections and 10.0 to 40.0 mL for intravenous injections, depending on the dose cohort.

#### Treatment

Patients received multiple doses of TILT-123: an intravenous dose on day 1 and intratumoral doses on days 8, 22, 36, 48, and 64. Patients judged as possibly benefiting could continue to receive additional rounds of TILT-123 beyond the primary endpoint. The intravenous dose ranged from 3×10^9^ to 4×10^12^ viral particles (VP) and the intratumoral dose ranged from 3×10^9^ to 5×10^11^ VPs according to the dose-escalation scheme. Dose-limiting toxicity (DLT) was defined as a toxicity preventing administration of the agent at that dose. Intratumoral injections were performed with ultrasound guidance using a 21-gauge needle. At least one tumor was injected during intratumoral dosing, with the agent distributed evenly to multiple locations inside each injected tumor.

### Outcomes

The primary objective of the study was to assess the safety of TILT123 by adverse events (AE), laboratory values, vital signs, and electrocardiograms. Secondary objectives included assessing the antitumor efficacy of TILT-123 by imaging, measuring immune responses against the tumor and the virus using different biological samples, and virus persistence in blood and its shedding.

### Laboratory measurements and correlative analyses

A complete blood count as well as liver and kidney tests were conducted by routine laboratory testing. AEs were graded according to Common Terminology Criteria for AEs (CTCAE) version 5.0.

Samples collected for correlative analyses during the trial included biopsies on baseline, day 8 pre-treatment and day 36 pre-treatment. Serum was collected on each treatment day pre-treatment and 16 hours post-treatment. Blood for clinical laboratory tests was collected pretreatment and 24 to 48 hours post-treatment. Urine, feces, saliva, and blood were collected for virus detection by qPCR.

Anti-adenovirus antibodies were measured by neutralizing antibody assay described in more detail previously, and a titer of 1:64 was the lowest assayed titer (18). qPCR to detect TILT-123 was conducted with primers targeting the viral transgene IRES-hIL2 region. Proteomic analysis of serum was conducted with Olink Immuno-Oncology assay. Tumor biopsies were fixed in formalin, processed to paraffin blocks and sectioned for multiplexed IHC staining with antibodies listed in Supplementary Table S1.

### Assessment of antitumor efficacy, survival and progressionfree survival

Antitumor efficacy was assessed on day 78 with contrastenhanced CT imaging and PET with ^18^F-FDG. Maximum tumor diameters and SUVmax readings were obtained from the images by a specialized radiologist. Tumor responses were evaluated by RECIST 1.1, iRECIST and PET-based criteria shown in Supplementary Table S2 (19). Disease control was defined as stable disease or better for RECIST 1.1 and iRECIST, and stable metabolic disease or better for PET-based criteria.

For individual lesion analysis, CT disease control was defined as lesion size increase less than 20%. For individual lesion PET analysis, metabolic disease control was defined as lesion SUVmax increase less than 30%.

Survival and progression-free survival (PFS) data were retrieved from the electrical clinical trial system. Data cutoff for survival, PFS, and time to progression (TTP) was November 26, 2023. Patients not reported as deceased or progressing at the data cutoff date were included in analysis as alive or non-progressed until the data cutoff date.

### Statistical analyses

All patients enrolled in the study were evaluated for safety. As no DLTs occurred, the final number of participants was 20 according to standard regulatory practices. For grouped analyses, two-sided *t* tests or Mann–Whitney *U* tests were used to compare groups, as reported in figure legends. For overall survival (OS) and grouped analyses, the Mantell–Cox Log-rank test or MaxCombo test was used to compare groups, as reported in figure legends. For categorical analyses, Fisher’s exact tests were used to compare groups. GraphPad Prism 9.4.1 and Rpackages “OlinkAnalyze” and “nph” were used for statistical analyses.

### Role of funding source

TILT Biotherapeutics Oy was involved in the study design, data analysis and interpretation, writing and submission of the report for publication.

### Data availability

Data presented in this study may be requested from the corresponding author. Data requests are subject to local laws, trial ethical board regulations, and the data sharing policy of TILT Biotherapeutics Oy.

## Results

### Patients

Thirty patients with advanced solid cancers were assessed for eligibility, of which 20 patients were enrolled into the trial. Baseline patient demographics are reported in Table 1. The most common cancer types included sarcomas (*N* = 7; 35%), melanomas (*N* = 3; 15%), and ovarian cancers (*N* = 3; 15%). The median age of patients was 58 years (range 33–72) and 65% of the patients were female. The median number of previous treatment lines was 4.5 and 25% of the patients had received ICIs previously. A total of 25% of patients had a WHO/ECOG performance score of 0 whereas 75% had a score of 1. Full patient demographics are presented in Supplementary Table S3.

**Table 1.** Patient demographics.

	Median (range) or *n*
Age, *y*	58 (33-72)
Sex, *n*	
Female	13
Male	7
Tumor type, *n*	
Sarcomas^a^	7
Melanomas^b^	3
Ovarian and peritoneal cancers	3
Head and neck cancers	2
Anaplastic thyroid carcinoma	1
Breast carcinoma, HER2^+^, ER^−^, PR^−^	1
Mucinous carcinoma—appendix	1
Neuroendocrine carcinoma—bladder	1
Non–small cell lung cancer, EGFR^−^, ALK^−^	1
Performance status (ECOG), *n*	
0	5
1	15
Number of previous systemic treatments, *n*	4.5 (1–15)
Time from diagnosis, months	60 (9–145)
Previous ICI, *n*	5

a Sarcomas included leiomyosarcomas (3), myxoid liposarcomas (2), chondrosarcoma (1), and rhabdomyosarcoma (1).

b Melanomas included cutaneous melanomas (2) and melanoma of unknown primary (1).

### Safety and pharmacokinetics

**Figure 1.**
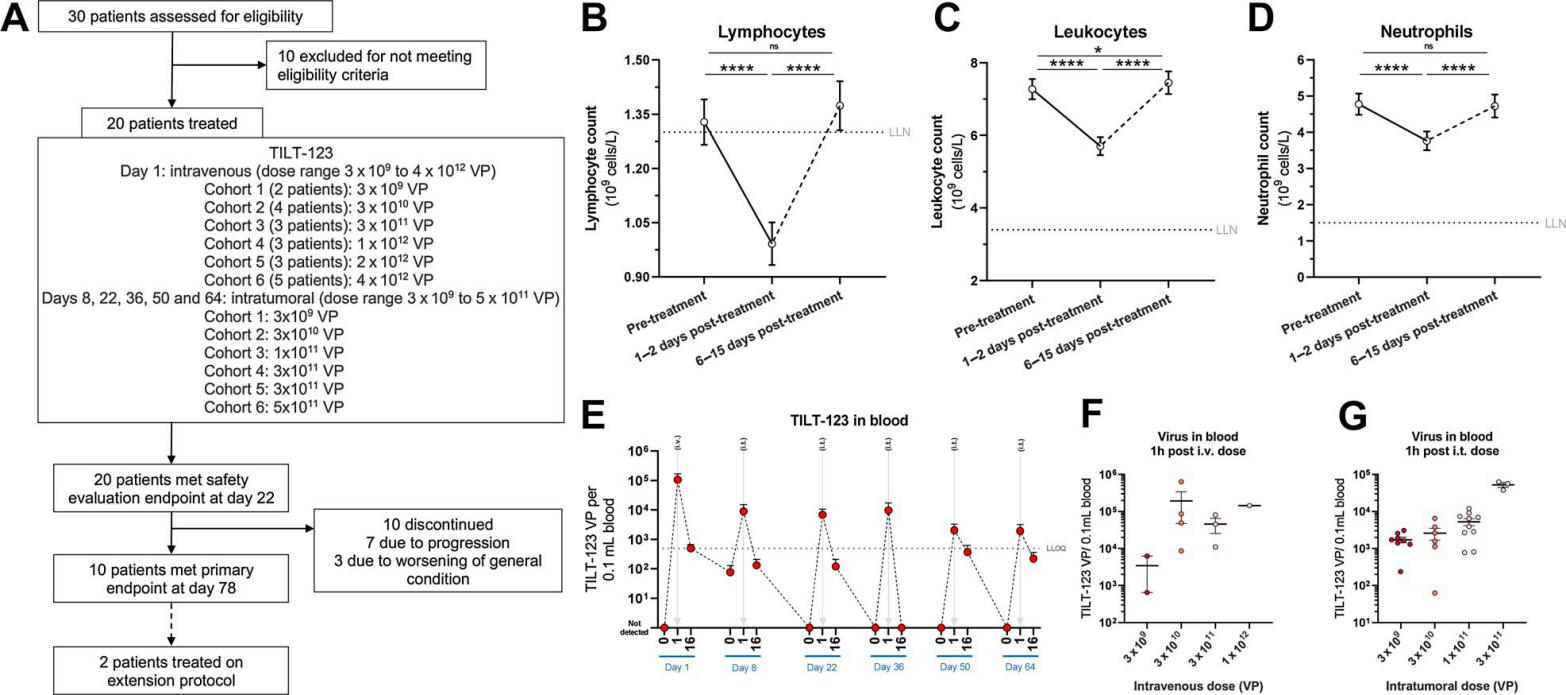
**A**, Trial outline. **B**, Lymphocyte changes after TILT-123, all cohorts pooled. **C**, Leukocyte changes after TILT-123, all cohorts pooled. **D**, Neutrophil changes after TILT-123, all cohorts pooled. **E**, Virus detection in blood by qPCR, before treatment, 1 hour post-treatment and 16 hours post-treatment, all dose cohorts available pooled (*N =* 10 patients). **F**, Virus detection in blood by qPCR 1 hour after intravenous dose, stratified by intravenous dose given. **G**, Virus detection in blood by qPCR 1 hour after intratumoral dose, stratified by intratumoral dose given. For all graphs, mean ± SEM shown. For **B–D**, *P* value from two-sided *t* test shown for graphs. LLOQ, lower limit of quantification; ns, non-significant (*P* > 0.05); *, *P* < 0.05; ****, *P* < 0.001.

Patients received an intravenous dose of TILT-123 on day 1 (dose range 3×10^9^×4 10^12^ VPs) and intratumoral doses of TILT-123 on days 8, 22, 36, 50, and 64 (dose range 1×10^9^–5×10^11^ VPs; Fig. 1A). Of 20 patients enrolled, 10 completed the trial and 10 patients discontinued (Fig. 1A). The treatment was well tolerated, and no DLTs were observed. Patients exhibited short-term reduction of blood lymphocytes following therapy, where lymphocyte counts decreased to below the lower limit of normal (LLN; Fig. 1B). Lymphocyte counts quickly normalized to baseline at the next available blood sampling time point (6–15 days post-treatment). No clinically relevant changes in leukocytes or neutrophils were observed although a small decrease, with values remaining above LLN, was observed after TILT-123 administration (Fig. 1C and D).

The most common AEs related to TILT-123 included fever (16.5% of all AEs), chills (12.8%), and fatigue (8.3%; Table 2). Three grade 4 treatment-related events were observed. Grade 4 neutropenia was seen in one patient (20104) on dose level 4, where the patient’s neutrophils decreased below normal on day 9 following intratumoral therapy on day 8 (Supplementary Fig. S1A). By day 13, the neutrophil count had started to increase, and neutrophils normalized by day 22. Some decrease was again seen after the next injection but after day 50 values remained normal. Interestingly, grade 2 cytokine release syndrome (typically caused by strong T-cell activation) was also recorded on day 9 for this patient.

**Table 2.** Adverse events related to TILT-123 therapy as judged and reported by the investigator, stratified by grade, cohort, and unique patients with reported adverse event.^a^

Event type							Cohort	Cohort	Cohort	Cohort	Cohort	Cohort	
	Grade 1	Grade 2	Grade 3	Grade 4	Grade 5	Total	1 (2 patients)	2 (4 patients)	3 (3 patients)	4 (3 patients)	5 (3 patients)	6 (5 patients)	Unique patients
*Infection-likesymptoms*													
Fever	14	4	0	0	0	**18**	0	1	6	2	0	9	**9/20**
Chills	13	1	0	0	0	**14**	0	3	1	2	2	6	**7/20**
Subfebrile body temperature	3	0	0	0	0	**3**	0	0	1	2	0	0	**2/20**
Flu-like symptoms	1	0	1	0	0	**2**	0	0	0	1	0	1	**2/20**
*General*													
Fatigue	5	3	1	0	0	**9**	0	2	1	2	2	2	**7/20**
Tiredness	3	3	0	0	0	**6**	1	0	1	0	4	0	**3/20**
Nausea	1	1	0	0	0	**2**	0	0	0	0	0	2	**2/20**
Dry mouth	1	0	0	0	0	**1**	0	0	0	1	0	0	**1/20**
Headache	1	0	0	0	0	**1**	0	0	0	0	1	0	**1/20**
Hot flushes	1	0	0	0	0	**1**	0	0	0	1	0	0	**1/20**
Tinnitus	1	0	0	0	0	**1**	1	0	0	0	0	0	**1/20**
*Hematological*													
Neutrophil count decreased	0	1	4	1	0	**6**	0	0	1	5	0	0	**2/20**
Leukocyte count decreased	0	4	1	0	0	**5**	0	0	1	4	0	0	**2/20**
Platelet count decreased	1	1	0	1	0	**3**	0	0	0	1	2	0	**2/20**
Lymphocyte count decreased	0	0	1	1	0	**2**	0	0	0	0	1	1	**2/20**
*Renal*													
Creatinine increased	1	1	0	0	0	**2**	0	0	0	1	0	1	**2/20**
*Gastrointestinal* Diarrhea	1	1	0	0	0	**2**	0	1	0	0	0	1	**2/20**
Loss of appetite	2	0	0	0	0	**2**	0	0	0	0	1	1	**2/20**
Vomiting	2	0	0	0	0	**2**	0	0	0	0	0	2	**2/20**
*Cardiovascular*													
Edema in feet	1	0	0	0	0	**1**	1	0	0	0	0	0	**1/20**
*Musculoskeletal*													
Muscle pain	4	1	0	0	0	**5**	0	0	0	0	4	1	**3/20**
Joint pain	3	1	0	0	0	**4**	0	0	0	1	0	3	**2/20**
Pain in extremities	2	0	0	0	0	**2**	0	1	0	1	0	0	**2/20**
Muscle cramps	1	0	0	0	0	**1**	0	0	0	0	1	0	**1/20**
Worsening of pain in left knee	0	1	0	0	0	**1**	0	0	0	1	0	0	**1/20**
*Immunological*													
Cytokine release syndrome	1	1	0	0	0	**2**	0	0	0	1	0	1	**2/20**
*Local lesion symptoms*													
Pain in tumor	4	4	0	0	0	**8**	0	0	0	0	7	1	**3/20**
Infection in tumor	0	1	0	0	0	**1**	0	0	0	0	1	0	**1/20**
Itching in tumor	1	0	0	0	0	**1**	0	0	0	0	1	0	**1/20**
Swelling of metastases	1	0	0	0	0	**1**	1	0	0	0	0	0	**1/20**
*Total*	**68**	**29**	**8**	**3**	**0**	**109**	**4**	**8**	**12**	**26**	**27**	**33**	**18/20**
*Total per patient*	**3.4**	**1.5**	**0.4**	**0.2**	**0**	**5.5**	**2**	**2**	**4**	**8.7**	**9**	**6.6**	

a Events reported as of November 11, 2023.

The second grade 4 AE was thrombocytopenia, seen in one patient (20108) on dose level 5. Investigation of the case revealed pseudothrombocytopenia, that is, aggregation of thrombocytes in EDTA blood tubes. After blood draws were repeated in citrate tubes, low platelet counts were not seen (Supplementary Fig. S1B). The third and final grade 4 AE was lymphopenia occurring in one patient (20219) in dose level 6. The patient’s lymphocyte count was slightly below normal before dosing, and the count decreased further after intravenous TILT123, but returned to normal by the next dosing (Supplementary Fig. S1C). None of the three grade 4 AEs led to treatment discontinuation. No grade 5 AEs related to therapy were observed in the trial.

No signs of liver damage measured by alanine aminotransferase (ALT) were noted across trial, and the intravenous dose level did not correlate to levels of ALT (Supplementary Fig. S1D and S1E). Most AEs occurred in cohort 5, and no clear correlation of AEs with dose was observed, although there were numerically more AEs per patient in cohorts 4–6 than in cohorts 1–3 (Table 2). All AEs with more than one occurrence reported during the trial are shown in Supplementary Table S4.

TILT-123 was detected in blood 1 hour after intravenous and intratumoral dosing (Fig. 1E). High viral amounts were detected 1 hour after treatment regardless of treatment route. Viral amount generally fell below the limit of quantification 16 hours posttreatment. Interestingly, a patient (20211) with detectable virus in blood 7 days after intravenous treatment (Supplementary Fig. S2A) was among the longest survivors in the trial, with 377 days survival. This patient also showed a relatively low production of antiadenoviral antibodies, with no neutralization detected at baseline and a highest titer of 4096 (Supplementary Fig. S2B).

An increase in circulating virus 1 hour after administration was seen with increasing dose for both intravenous and intratumoral dosing (Fig. 1F and G). No virus was detected in urine or feces of any patient.

### Efficacy

**Figure 2.**
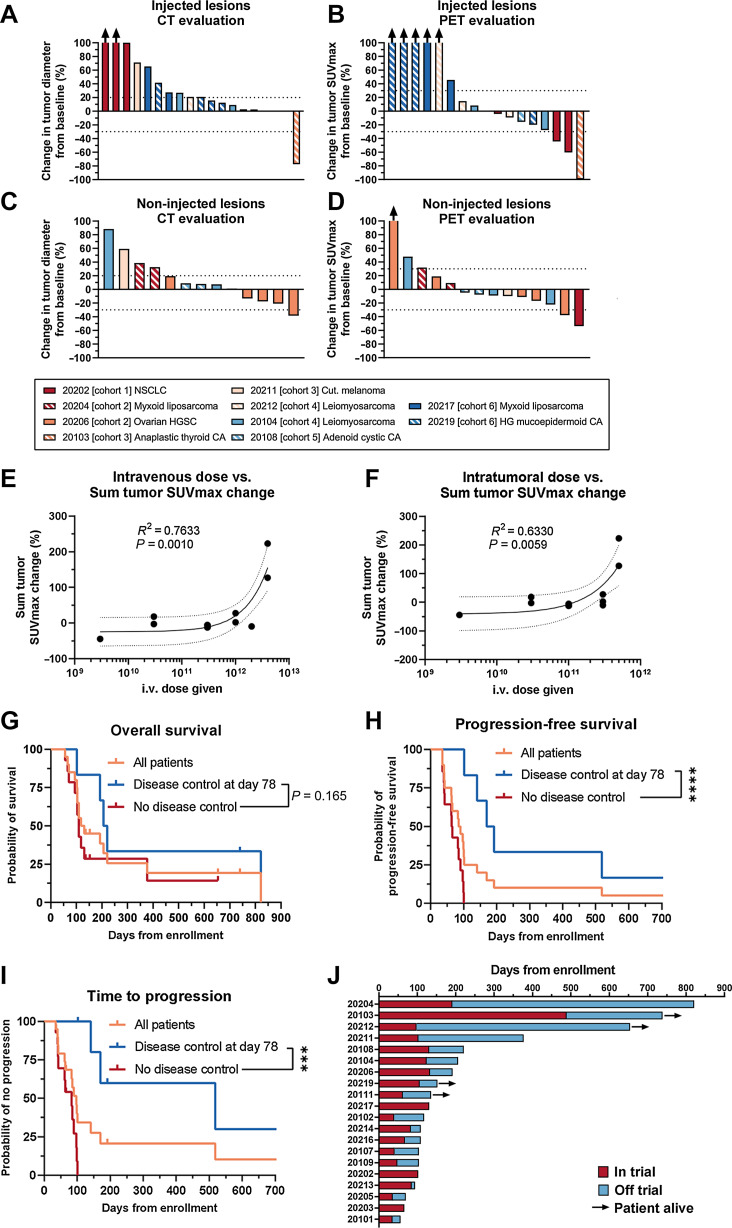
**A**, Response evaluation in all injected lesions, evaluated by CT. **B**, Response evaluation in all injected lesions, evaluated by PET. **C**, Response evaluation in allimaged non-injected lesions, evaluated by CT. **D**, Response evaluation in all imaged non-injected lesions, evaluated by PET. Best response shown for **A–D** if patient continued to extension. **E**, Intravenous dose given versus sum SUVmax change of measured lesions on day 78. **F**, Intratumoral dose given versus sum SUVmax change of measured lesions on day 78. **G**, Overall survival in the trial. **H**, Progressionfree survival in the trial. **I**, Time to progression in the trial. **J**, Swimmer plot of the patients enrolled in the trial. For **E** and **F**, linear fit shown with 95% confidence intervals shown with *R2* for goodness of fit and *P* value for slope deviation from zero. For **G–I**, disease control defined with PET-based criteria and comparison of disease control and no disease control evaluated with Mantel–Cox Log-rank test. ***, *P* < 0.001; ****, *P* < 0.0001.

Antitumor efficacy was seen in both injected and non-injected tumors. Injected and non-injected lesions were assessed on patients available for imaging on day 78 with CT and CT-PET (Fig. 2A–D). In injected lesions, disease control was seen in 9/19 lesions by CT and 11/17 by PET (Fig. 2A and B). Regarding non-injected lesions, disease control was seen in 9/13 lesions by CT and 11/14 lesions by PET (Fig. 2C and D). Regarding dose and treatment effect, no clear correlation to either intravenous or intratumoral dose was given to tumor diameter change (Supplementary Fig. S3A and S3B), but interestingly tumor SUVmax increase was positively correlated with increase in both intravenous and intratumoral dose given (Fig. 2E and F; *P* = 0.0010 and *P* = 0.0059, respectively). When comparing dose given and survival, no trend between dose and OS or PFS could be seen (Supplementary Fig. S3C–S3E, *P* = 0.5232 and *P* = 0.3242, respectively). However, when analyzing TTP, a trend for benefit with lower dose could be seen (Supplementary Fig. S3E, *P* = 0.0292).

Median survival of all patients enrolled in the trial was 124.5 days (Fig. 2G). For patients showing disease control, the median survival was 213.5 days whereas for patients not showing disease control the median survival was 109 days (*P* = 0.165, Fig. 2G). The median PFS of all patients enrolled was 87.5 days (Fig. 2H). For patients showing disease control at day 78, the median PFS was significantly longer at 181 days, compared with 65 days in patients not showing disease control at day 78 (Fig. 2H, *P* < 0.0001). The median TTP for all patients was 97 days (Fig. 2I). Patients showing disease control at day 78 had markedly longer median TTP at 518 days, compared with patients without disease control with median TTP of 83 days (Fig. 2I, *P* = 0.0002).

Of note, four patients showed markedly long survival, with three patients alive more than 600 days after enrollment (Fig. 2J). Four patients surviving more than 1 year after enrollment included a patient with myxoid liposarcoma (20204), anaplastic thyroid carcinoma (20103), leiomyosarcoma (20212), and nodular melanoma (20211). Notably, three of the four patients were heavily pre-treated and resistant to other therapies. Patient 20204 with myxoid liposarcoma had received 10 previous cancer therapies consisting of neo-adjuvant radiotherapy, 3 surgeries and 6 lines of chemotherapy. The patient completed the trial and received 4 additional rounds of TILT-123 intratumorally, before finally succumbing to the disease 821 days after enrollment in the trial. The patient did not receive other cancer therapies after the trial, aside from palliative radiotherapy to a groin metastasis. Therefore, the patient lived for more than 600 days after the trial without any further systemic therapies administered.

**Figure 3.**
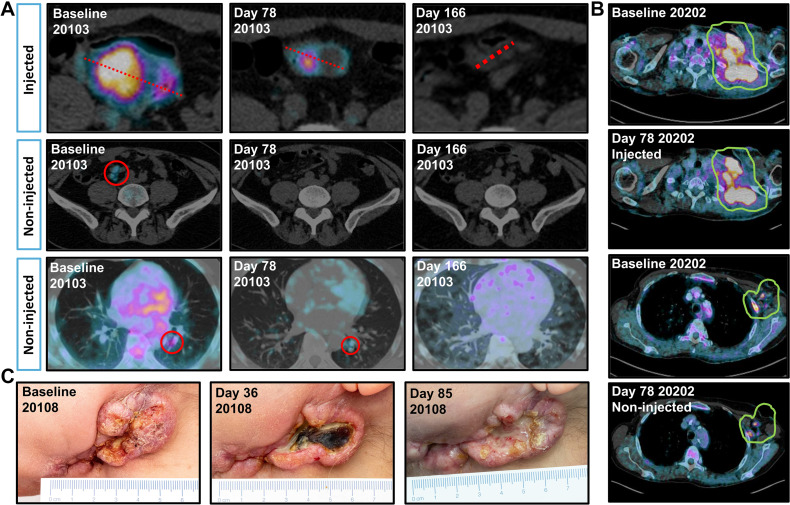
**A**, Volume and PET signal changes in patient 20103 with metastatic anaplastic thyroid carcinoma, showing disappearance of PET signal for injected abdominal lesion, and disappearance of mesenteric and pulmonal lesions by PET and CT. **B**, Changes in PET signal in patient 20202 with metastatic NSCLC, showing decrease in PET signal for injected (62% SUVmax decrease) and non-injected lesion (54% SUVmax decrease). **C**, Visual changes in tumor in patient 20108 with adenocystic adenocarcinoma of the head and neck, showing marked necrosis of the tumor post-treatment.

Patient 20103 with anaplastic thyroid carcinoma had received surgery with adjuvant radiotherapy, and paclitaxel combined with radiotherapy in the metastatic setting before enrollment in the trial. Shrinkage of injected and non-injected tumors was seen in CT imaging of the patient, which was evaluated as a partial response by RECIST 1.1 (Fig. 3A). The patient received 4 additional rounds of intratumoral TILT-123 and 3 additional rounds of intravenous TILT-123, followed by off-trial radiotherapy and pembrolizumab, and is alive at the time of writing 739 days after trial enrollment. The patient’s tumor was microsatellite stable with high tumor mutational burden.

Patient 20212 with leiomyosarcoma had received 6 regimens of previous cancer therapy consisting of 2 radiotherapy regimens and 4 lines of chemotherapy. The patient completed the trial and was evaluated as having progressive disease at day 78. The patient received palliative doxorubicin and pazopanib after the trial, and is alive at the time of writing of the article 654 days after enrollment.

The fourth patient with long survival was a patient with nodular melanoma (20211). The patient was markedly treatment resistant, having had 4 rounds of surgery, 2 lines of nivolumab, paclitaxel combined with carboplatin and an investigational checkpoint inhibitor BMS-986218 targeting CTLA-4. The patient was evaluated as having progressive disease on day 78, and received palliative temozolomide and radiotherapy after the trial. The patient survived 295 days after the last dose of TILT-123.

Efficacy in a checkpoint inhibitor resistant setting was also seen in patient 20202 with EGFR and ALK negative non–small cell lung cancer (NSCLC) refractory to nivolumab therapy. The patient showed a reduction in PET signal in both the injected neck lesion (62% SUVmax decrease) and non-injected axillar metastases (54% SUVmax decrease; Fig. 3B). Patients with previous ICI therapy showed comparable changes in tumor diameters and PET activity (Supplementary Fig. S4A–S4D) and time-to-event analyses (Supplementary Fig. S4E– S4G) when compared with ICI-naї1ve patients.

Long survival of several patients whose imaging evaluations were unfavorable (Fig. 2A–D and J) suggests that the imaging and clinical criteria used were not able to capture treatment efficacy in all cases. Strong inflammation is likely to enlarge tumors in a phenomenon called pseudoprogression, resulting in patients being taken off the trial, even if the therapy is working as planned (20). Furthermore, photographs of patient 20108 demonstrate why imaging criteria can be suboptimal. Although the treatment caused notable tumor necrosis, followed by signs of scarring (Fig. 3C), tumor size was not much impacted. Size based criteria such as RECIST 1.1 and iRECIST are particularly sensitive to pseudoprogression, and whereas FDG PETbased criteria may be somewhat better, activated lymphocytes consume a lot of sugar, again raising the possibility of false positives and missed detection of beneficial treatment effects (19).

### Pharmacodynamics and biopsy analysis

**Figure 4.**
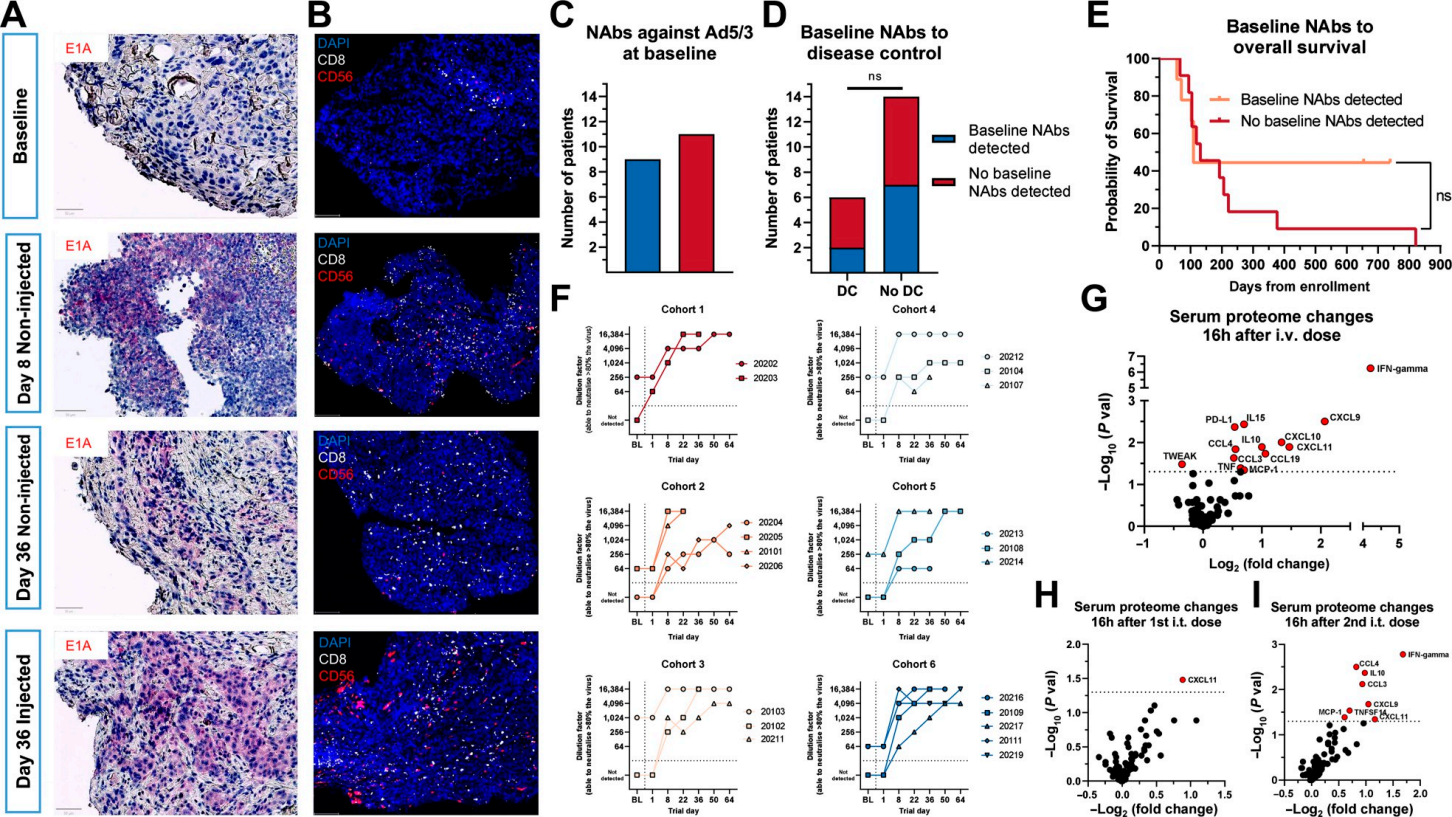
**A**, Virus staining (violet) in tumor biopsies from patient 20202 at different time points across the trial, showing productive virus replication in injected and noninjected lesions; scale bar, 50 mm. **B**, Tumor IHC from patient 20202 staining for DAPI, CD8, and CD56 showing increased numbers of effector lymphocytes in injected and non-injected lesions post-treatment; scale bar, 100 μm. **C**, Neutralizing antibodies (NAbs) against TILT-123 at baseline in all patients. **D**, Neutralizing antibody presence compared with disease control at day 78. Disease control defined as SMD or better at day 78. No disease control defined as PMD or NA at day 78. Groups compared with the Fisher’s exact test. **E**, Baseline neutralizing antibody presence compared with overall survival across trial. Groups compared with the MaxCombo log-rank test. **F**, Neutralizing antibody titer across trial in all dose cohorts. Baseline defined as day 1 pre-treatment value, for days 1 to 64 the highest titer shown for each day (pre-or post-treatment). **G**, Serum proteomic changes 16 hours after intravenous dosing of TILT-123, pooled patients from cohorts 1 to 5 (*n* = 15). **H**, Serum proteomic changes 16 hours after first dose of intratumoral dosing of TILT-123, pooled patients from cohorts 1 to 5 (*n* = 15). I, Serum proteomic changes 16 hours after second dose of intratumoral dosing of TILT-123, pooled patients from cohorts 1 to 5 (*n* = 15). For **G–I**, difference between pre‐and post-treatment protein calculated with the Mann–Whitney *U* test; ns, non-significant (*P* > 0.05).

We aimed to study the inflammatory processes after TILT-123 by using serial biopsies from patients across the trial. An example of this is seen in patient 20202 with metastasized NSCLC. Virus was detected in injected and non-injected lesions by IHC, supporting the notion of systemic spreading of the virus (Fig. 4A). At baseline, the assessed tumors had low infiltration by effector lymphocytes, characteristic of immunotherapy resistant “cold” tumors (Fig. 4B). After intravenous TILT-123 treatment, an increase in CD8^+^ and CD56^+^ cells was seen (Fig. 4B). This effect was further enhanced following intratumoral dosing (Fig. 4B).

### Immunostimulatory effects

At baseline, 9/20 patients had a low or no neutralizing antibody titer against TILT-123 (defined as titer less than 1:64), whereas 11/20 patients had detectable titers (Fig. 4C). No patients had high titers at baseline, consistent with the lack of a 5/3 chimeric adenovirus in nature. The baseline level of neutralizing antibodies did not associate with disease control (Fig. 4D), but patients with baseline neutralizing antibodies presented a tail of long-term survivors although not reaching significance due to small sample size (Fig. 4E, *P* = 0.258). In addition, the two best responders according to RECIST 1.1 (20103 and 20108) both developed the highest measurable titer of neutralization (Supplementary Fig. S5A). Similarly, both best PET responders and 2/4 longest survivors developed the highest measurable titer of neutralization (Supplementary Fig. S5B and S5C). Neutralizing antibody titers increased in all dose cohorts across trial without clear association to dose (Fig. 4F).

Systemic pro-inflammatory changes in the serum proteome were noted, with intravenous delivery inducing high fold changes of circulating IFNγ, CXCL9, and CXCL10 (Fig. 4G). Regarding intratumoral dosing, small changes in the proteome were noted after the firstintratumoral dosing, but more pronounced effects appeared after the second intratumoral dose, including pro-inflammatory markers similar to those observed in intravenous dosing, indicating a possible advantage of repeated intratumoral dosing (Fig. 4H and I). Both IL2 and TNFa increased in the serum after intravenous and intratumoral dosing (Supplementary Fig. S6A and S6B).

## Discussion

Most solid cancers still remain incurable with immunotherapy due to marked power imbalance between infiltrating pro-tumor and antitumor cells, often presenting as a lack of tumor-infiltrating lymphocytes (TIL; “cold tumors”; refs. 1, 21). Oncolytic viruses are emerging as a treatment modality to shift this balance to favor efficient anticancer immune responses (22). Previously, oncolytic viruses have shown efficacy compatible with regulatory approval in select solid cancers, namely, melanoma, glioma, and squamous cell carcinoma of the head and neck (SCCHN; refs. 23–26). Also, there is a recent approval of a non-replicating adenovirus coding for IFN alpha administered locally into the bladder, underlining the potential of armed adenoviruses in immunotherapy (27). However, currently approved oncolytic virus therapies are limited to intratumoral administration, restricting their widespread use. Also, earlier generation agents encode no transgenes or produce transgenes such as GMCSF that can also induce immunosuppressive effects through myeloid-derived suppressor cells.

TILT-123 is based on a serotype 5 adenovirus, with a 24 kb deletion in the E1A region and addition of the E2F promoter to facilitate cancer cell–specific replication (2). The fiber knob of the virus has been changed to a serotype 3 knob to enhance systemic delivery and cancer cell entry (5, 6, 28). TILT-123 carries two immunostimulatory transgenes: TNFα and IL2 (2).

The transgenes in TILT-123 were chosen by preclinical comparison of a long list of molecules potentially able to enhance T-cell infiltration, proliferation, and cancer cell killing (3, 4, 15). TNFα is a potent proinflammatory protein, and acute signaling through TNFR1 produces pro-inflammatory and pro-apoptotic changes in the tumors, whereas short-term signaling through TNFR2 expressed on lymphocytes leads to cell proliferation (29, 30). On the other hand, IL2 is recognized as one of the key cytokines for T-cell proliferation and is considered as the first form of immunotherapy (31, 32). Thus, TNFa was incorporated into TILT-123 to induce danger signaling and promote lymphocyte trafficking into tumors with IL2 to promote the activation of the trafficked lymphocytes and support their proliferation (2, 3, 15, 33). Both transgenes work synergistically with oncolytic adenovirus therapy due to the lymphocyte dominated immune response that occurs in humans after oncolytic adenovirus therapy (11, 34). TILT123 continues to be one of the few clinically used oncolytic viruses that target the T-cell compartment specifically.

The appealing attributes of TNFα and IL2 have led to their utilization in oncology as recombinant proteins and vectored form. Recombinant TNFα is used in isolated limb perfusion for the treatment of soft tissue sarcomas (35). Vectored delivery of TNFα culminated in TNFerade, a replication incompetent adenoviral vector, which was studied in a randomized phase 3 trial in combination with fluorouracil and radiotherapy, for the treatment of locally advanced pancreatic cancer. Although promising in phase I/II trials, the phase III trial of TNFerade failed to produce a survival benefit compared with standard of care (36). The failure of TNFerade was thought to arise from poor transduction of tumor cells. Pancreatic tumors are notoriously difficult to inject in ultrasound guidance, and the TNFerade approach required not only the virus to reach tumor cells, but also the subsequent radiotherapy needed to hit the same cells to activate the promoter driving TNFα expression. Although oncolytic viruses can locally amplify, non-replicating agents such as TNFerade require extreme delivery precision. These difficulties were compounded by the challenging patient population (36).

IL2 has been used as a cancer immunotherapeutic since the 1980s, and can provide long-term responses, and even cures, in some melanoma and renal cell cancer patients (31). IL2 is also used as a companion therapeutic to enhance the efficacy of adoptive cell therapies such as tumor infiltration lymphocyte therapy (37). However, systemic administration of IL2 is limited by severe off-target toxicities, most commonly vascular leak syndrome (38). These AEs restrict the doses that can be used, which in turn results in sub-optimal intratumoral concentrations, limiting efficacy (38). Of note, clinical trials conducted in the early 2000s showed promise of vectored delivery of IL2 (39).

Our work here describes the results from TUNIMO, a monotherapy dose-escalation phase I trial with TILT-123. The trial confirmed that TILT-123 treatment is safe and able to produce proinflammatory changes at tumors even in this difficult to treat patient population. Notably, antitumor activity and long survival times were seen in several patients that had failed multiple rounds of previous therapy. Discrepancy between observed long survival times and treatment efficacy measured by CT stems from the challenge of capturing treatment effect with imaging in immunooncological studies (40–42). This trial used PET-imaging alongside CT to capture treatment effect. A possible addition to enhance capturing of treatment effect could be the utilization of singlephoton emission CT (SPECT). SPECT has successfully been used in preclinical and clinical settings to capture treatment efficacy of immunotherapeutics, including oncolytic adenoviruses (43, 44). However, further research is needed to optimize SPECT imaging for clinical trial application.

A remarkable phenomenon observed in patients dosed with TILT123 was the transient decrease in lymphocytes 1 to 2 days after therapy, without a similar magnitude of change in all leukocytes or neutrophils. Accompanied by the increase of CD8^+^ and CD56^+^ lymphocytes in the tumors, these findings are suggestive of immune cell trafficking from the periphery to the tumors, turning “cold” tumors “immunologically hot.”

Antitumor effects and lymphocyte infiltration were seen in both injected and non-injected lesions, demonstrating systemic effects of treatment. No clear correlation of dose to OS or PFS was observed, although a trend for longer TTP was seen with lower dose levels. Some patients showed durable viral circulation even 7 days after intravenous delivery, most likely due to production of VPs in tumors and release to the systemic circulation.

Virus was seen in both injected and non-injected lesions by IHC. Therefore, as seen in preclinical studies, the systemic effects of the therapy are mediated by two mechanisms: systemic dissemination of the virus and systemic immune response (16). The IHC marker used for viral detection included the viral E1A protein, which is expressed in the early part of the viral replication cycle. This protein is not present in mature virions; thus the E1A protein is only seen when the virus replicates and so the detected E1A viral protein suggests active replication of TILT-123 inside the tumors. Of note, both virus and accompanying immunological changes could be detected in tumors already 7 days after an intravenous injection, before any intratumoral injections. This suggests that TILT-123 is able to transduce tumors through the intravenous route.

Neutralizing antibodies have been assumed to be a limiting factor for oncolytic virotherapy, especially highly immunogenic vectors such as Ad5. However, reports from animals and patients have challenged this hypothesis (45–47). It has been proposed that the neutralizing antibody response, which has evolved to neutralize small amounts of virus entering systemic circulation following epithelial replication, cannot fully neutralize the large amounts of oncolytic virus present in blood after intravenous or intratumoral injection. In addition, some viruses, such as 5/3 chimeric adenovirus (e.g., TILT-123), can use cells of the blood to partially bypass neutralization (48). Furthermore, a mounting body of evidence suggests that immunity arising against oncolytic virus agents is a multifaceted phenomenon, and that antiviral immunity can in fact enhance the anti-cancer effects of oncolytic viruses through epitope spreading, antibody-mediated cytotoxicity and complement activation (14, 49). In the present study, we showed that all patients developed an increase in the amount of neutralizing antibodies against the virus, but baseline-neutralizing antibody titer was not correlated with efficacy and seropositive patients at baseline had a trending benefit in OS. Furthermore, even though most patients developed high neutralizing titer against the vector, virus was detectable in the blood and biopsies after intravenous and intratumoral administration. As seen in previous publications, oncolytic adenoviruses are able to circulate systemically and transduce distant metastases even in the presence of neutralizing antibodies (45). A possible mechanism behind the advantageous “escape” from neutralizing antibodies can be different pharmacokinetics of viral vectors as compared with neutralizing antibodies: Viral vectors can use an active form of transport through cell surface receptors to enhance tumor penetration, whereas antibodies rely on passive diffusion from blood to tumors.

Pro-inflammatory changes were observed in the serum after intravenous and intratumoral administration. Notably, high levels of chemokines associated with T-cell trafficking (CXCL9, CXCL10, and CXCL11) and T-cell cytotoxicity (IFNγ and TNFα) were seen after intravenous and intratumoral administration. Proteomic changes seen after the intravenous dosing of TILT-123 are likely tied to natural antiviral responses (50). Curiously, the first intratumoral dosing of TILT123 did not elicit strong serum proteomic changes aside from small upregulation of CXCL11. However, after the second intratumoral dose of TILT-123, a stronger serum proteomic signal was seen with upregulation of IFNγ and multiple chemokines (CXCL9, CXCL11, CCL3, and CCL4).

In conclusion, treatment with TILT-123 was safe and able to produce favorable clinical and immunological antitumor effects in this difficult to treat patient demographic. Systemic viral circulation was seen after intravenous and intratumoral dosing. Virus and antitumor effects were seen in both injected and non-injected lesions, following either intravenous or intratumoral delivery. Keeping in mind that TILT-123 was developed for activating T cells, it will be interesting to see the results of combination therapies with synergistic approaches such as checkpoint inhibitors and adoptive cell therapy with TILs. The good safety profile of TILT-123 monotherapy seen in this phase I trial facilitates such combination approaches and several trials are ongoing (NCT04217473, NCT05271318, NCT05222932, and NCT06125197).

## Supplementary Material

Supplementary Data 1 Supplementary Table S1-S5, Supplementary Figure S1-S6
