# Supplementary material for: Safety, Efficacy, and Biological Data of T-Cell–Enabling Oncolytic Adenovirus TILT-123 in Advanced Solid Cancers from the TUNIMO Monotherapy Phase I Trial
Source: Clin Cancer Res. 2024 Mar 27;30(17):3715–25. doi: 10.1158/1078-0432.CCR-23-3874 (PMC11369615; doi:10.1158/1078-0432.CCR-23-3874)
Supplement: Supplementary Data 1 — Supplementary Table S1-S5, Supplementary Figure S1-S6 [file ccr-23-3874_supplementary_data_1_suppds1.pdf]

Antibody	Clone	Catalog	Manufacturer
Adenovirus-5 E1a	M58	sc-58658	Santa Cruz Biotechnology
CD56	MRQ-42	156R-94	Cell Marque
CD8	C8/144B	M7103	Dako
Ecadherin	24E10	CST 3195	Cell Signaling Technology
Cytokeratin		ab9377	Abcam

Supplementary Table 1.

PET Criteria	
Complete metabolic response (CMR)	Complete resolution of FDG activity within measurable lesions and all reliably assessable lesions to background levels. No new FDG-avid lesions in pattern typical of cancer*. Lymph nodes may remain metabolically active due to immune response (activated lymphocytes take up FDG).
Partial metabolic response (PMR)	> 30 % reduction in FDG activity measured as the summed SUVmax of measurable lesions (up to five lesions, max 2/organ). No new FDG-avid lesions in pattern typical of cancer*.
Minor metabolic response (MMR)	10-29 % reduction in FDG summed SUVmax. No new FDG-avid lesions in pattern typical of cancer*.
Stable metabolic disease (SMD)	0-9 % reduction or up to < 30 % increase in FDG summed SUVmax. No new FDG-avid lesions in pattern typical of cancer*.
Progressive metabolic disease (PMD)	≥ 30 % increase in FDG summed SUVmax in pattern typical of tumor, or new clearly FDG-avid clinically significant lesions in pattern typical of cancer*. Increase in metabolic activity in lymph nodes should not result in PMD if no progression is detected elsewhere, since it might reflect immunological activation and not progression.
*"clinically significant lesions in pattern typical of cancer" are defined as lesions associated with a CT abnormality most consistent with cancer (≥ 2 cm in diameter), and clearly not because of inflammation of infection or related to treatment response.	

Supplementary Table 2.

Patient ID	Cohort	VP Dose i.v./i.t.	Age, years	Sex	ECOG	Tumor type	Additional tumor info	Stage	Previous treatments	No. of prior systemic treatments	Prior ICI	Time from diagnosis, months	RECIST 1.1 at end of trial (day 78 or extension)	iRECIST at end of trial (day 78 or extension)	PET evaluation at end of trial (day 78 or extension)	Progression-free survival days
20202	1	$3x10^{9}$/ $3x10^{9}$	65	F	1	NSCLC	EGFR-, ALK-, PDL1 60%	IV	Cisplatin + Pemetrexed, Pemetrexed, Radiation, Pembrolizumab, Nivolumab, Nab-paclitaxel	5	Yes	18	PD	iUPD	PMR	102
20203	1	$3x10^{9}$/ $3x10^{9}$	60	F	1	Melanoma of unknown primary	No primary tumor found	IV	Radiation, Ipililumab + Nivolumab, Dabrafenib, Trametinib, Radiation, Radiation, Temozolomide, Pembrolizumab, Radiation, Surgery, Temozolomide + Vincristine + Lomustine, Paclitaxel + Carboplatin	7	Yes	17	N/A	N/A	N/A	66
20204	2	$3x10^{10}$/ $3x10^{10}$	50	M	0	Myxoid liposarcoma		IV	Radiation, Surgery x 2, Doxorubicin + Ifosfamide, Surgery, Eribulin, Trabectedin, Eribulin, Gemcitabine + Docetaxel	5	No	63	PD	iUPD	SMD	518
20205	2	$3x10^{10}$/ $3x10^{10}$	64	F	1	Ovarian serous cystadenocarcinoma		IV	Surgery, Adjuvant Paclitaxel + Carboplatin, Surgery, Paclitaxel + Carboplatin, Neoadjuvant Paclitaxel + Carboplatin, Surgery, Pegylated liposomal doxorubicin, Carboplatin, Paclitaxel, Gemcitabine, Pegylated liposomal doxorubicin, Letrozole, Gemcitabine + Bevacizumab, Cisplatin, Topotecan, Cisplatin, Etoposide	14	No	103	N/A	N/A	N/A	36
20101	2	$3x10^{10}$/ $3x10^{10}$	52	F	1	Breast carcinoma	HER2+, ER-, PR-	IV	Surgery, Trastuzumab + Docetaxel, CEF, Trastuzumab, Trastuzumab + Pertuzumab + Docetaxel, Lapatinib + Capecitabine, Paclitaxel + Carboplatin, Radiation, CEF, Radiation x 2	7	No	73	N/A	N/A	N/A	35
20206	2	$3x10^{10}$/ $3x10^{10}$	71	F	1	High grade serous ovarian carcinoma		IIIC	Surgery, Paclitaxel + Carboplatin, Bevacizumab, Docetaxel x 2, Pegylated liposomal doxorubicin, Carboplatin, Pegylated liposomal doxorubicin + Carboplatin, Gemcitabine, Topotecan, Etoposide, Docetaxel, Vinorelbine, Cisplatin, Radiation, Letrozol, Radiation	16	No	83	PD	iUPD	SMD	192
20103	3	$3x10^{11}$/ $1x10^{11}$	47	M	0	Anaplastic thyroid carcinoma		IV	Surgery, Radiation, Paclitaxel, Radiation	1	No	9	PR	iPR	PMR	739
20102	3	$3x10^{11}$/ $1x10^{11}$	38	M	1	Rhabdomyosarcoma		IV	Surgery, Radiation, Vincristine + Dactinomycin + Cyclophoshamide, Radiation, Vinorelbine, Pazopanib, Surgery x 2	3	No	28	N/A	N/A	N/A	40
20211	3	$3x10^{11}$/ $1x10^{11}$	66	F	1	Cutaneous melanoma	Nodular	IV	Surgery x 3, Nivolumab, Surgery, Nivolumab, Paclitaxel, Carboplatin, BMS-986218	3	Yes	48	PD	iUPD	PMD	97
20212	4	$1x10^{12}$/ $3x10^{11}$	63	F	1	Leiomyosarcoma	Subcutaneous	IV	Doxorubicin + Ifosfamide, Radiation, Docetaxel + Gemcitabine, Radiation, Docetaxel + Gemcitabine x 2	4	No	73	PD	iUPD	PMD	90
20104	4	$1x10^{12}$/ $3x10^{11}$	56	M	1	Leiomyosarcoma	Renal	IV	Surgery, Doxorubicin + Ifosfamide x 2, Gemcitabine + Docetaxel, Trabectedin, Pazopanib, Gemcitabine + Docetaxel, Radiation x 5	6	No	78	PD	iUPD	SMD	170
20107	4	$1x10^{12}$/ $3x10^{11}$	51	M	0	Chondrosarcoma	Neck	IV	Surgery, Radiation, Doxorubicin + Ifosfamide, Gemcitabine + Docetaxel	2	No	35	N/A	N/A	N/A	41
20213	5	$2x10^{12}$/ $3x10^{11}$	66	M	1	Neuroendocrine carcinoma of the bladder		IV	Surgery, Carboplatin + Etoposide, Capecitabine + Temozolomide, Radiation x 2, Carboplatin + Gemcitabine	3	No	13	N/A	N/A	N/A	85
20108	5	$2x10^{12}$/ $3x10^{11}$	52	F	1	Adenoid cystic carcinoma	Face and sinuses	IV	Surgery, Radiation, Surgery, Adjuvant Radiation, Surgery x 2, Radiation	0	No	145	SD	iSD	SMD	141
20214	5	$2x10^{12}$/ $3x10^{11}$	72	F	1	Mucinous carcinoma of the appendix		IV	Capecitabine, Surgery, Oxaliplatin + Capecitabine + Bevacizumab, Capecitabine + Irinotecan + Bevacizumab, Surgery, Regorafenib, Radiation, Regorafenib, Trifluridine/tipiracil	6	No	59	N/A	N/A	N/A	83
20216	6	$4x10^{12}$/ $5x10^{11}$	33	F	1	Cutaneous melanoma	Nodular	IV	Surgery x 3, Pembrolizumab, Surgery, Ipilimumab + Nivolumab, Temozolomide, Paclitaxel + Carboplatin, Trametinib	2	Yes	71	N/A	N/A	N/A	42
20109	6	$4x10^{12}$/ $5x10^{11}$	61	F	0	Leiomyosarcoma	Uterine	IV	Surgery, Gemcitabine + Docetaxel, Pazopanib, Radiation, Trabectedin, Radiation, Letrozole, Doxorubicin	5	No	23	N/A	N/A	N/A	64
20217	6	$4x10^{12}$/ $5x10^{11}$	51	F	1	Myxoid liposarcoma		IV	Surgery, Radiation, Surgery, Doxorubicin + Ifosfamide, Trabectidin x 2, Eribulin	4	No	61	PD	iUPD	PMD	101
20219	6	$4x10^{12}$/ $5x10^{11}$	42	M	1	High grade mucoepidermoid carcinoma of the parotid gland		IV	Surgery x 3, Radiation, Paclitaxel + Carboplatin, Cisplatin + Fluorouracil	2	No	39	PD	iCPD	PMD	99
20111	6	$4x10^{12}$/ $5x10^{11}$	63	F	0	High grade serous carcinoma of the peritoneum		IV	Paclitaxel + Carboplatin, Gemcitabine + Carboplatin, Surgery, Gemcitabine + Carboplatin x 3, Carboplatin x 3, Pegylated Liposomal Doxorubicin, Topotecan, Letrozole, Radiation, Carboplatin, Olaparib, Carboplatin, Paclitaxel, Docetaxel, Gemcitabine, Etoposide, Vinorelbine, Cisplatin, Atezolizumab, Tamoxifen, Radiation, Doxorubicin + Bevacizumab	15	Yes	123	N/A	N/A	N/A	62

Supplementary Table 3.

Event type	Grade 1	Grade 2	Grade 3	Grade 4	Grade 5	Total
Infection like symptoms						
Fever	16	4	0	0	0	**20**
Chills	13	1	0	0	0	**14**
Subfebrile body temperature	3	0	0	0	0	**3**
Cough	2	0	0	0	0	**2**
Flu like symptoms	3	0	1	0	0	**4**
General						
Fatigue	6	7	2	0	0	**15**
Nausea	5	4	0	0	0	**9**
Tiredness	4	3	0	0	0	**7**
Dyspnea	2	3	1	0	0	**6**
Worsening of general condition	0	1	0	0	4	**5**
Dizziness	1	1	0	0	0	**2**
Headache	2	0	0	0	0	**2**
Insomnia	2	0	0	0	0	**2**
Hematological						
Neutrophil count decreased	0	1	4	1	0	**6**
Leukocyte count decreased	0	4	1	0	0	**5**
Platelet count decreased	1	1	0	1	0	**3**
Lymphocyte count decreased	0	0	1	1	0	**2**
Hemoglobin count decreased	1	1	0	0	0	**2**
Renal						
Creatinine increased	1	1	0	0	0	**2**
Urological						
Dysuria	2	0	0	0	0	**2**
Gastrointestinal						
Diarrhea	2	2	0	0	0	**4**
Loss of appetite						
Vomiting	4	3	0	0	1	**8**
Abdominal pain	4	3	0	0	0	**7**
Abdominal swelling	2	0	0	0	0	**2**
Bleeding from rectum	1	1	0	0	0	**2**
Decreased appetite	2	0	0	0	0	**2**
Gastroenteritis	2	0	0	0	0	**2**
GI obstruction	0	1	1	0	0	**2**
Heartburn	1	1	0	0	0	**2**
Cardiovascular						
Edema in feet	5	3	0	0	0	**8**
Ascites	1	1	1	0	0	**3**
Tachycardia	0	2	0	0	0	**2**
Musculoskeletal						
Pain in extremities	1	4	2	0	0	**7**
Muscle pain	4	1	0	0	0	**5**
Joint pain	3	1	0	0	0	**4**
Muscle cramps	0	1	1	0	0	**2**
Muscle stiffness	2	0	0	0	0	**2**
Immunological						
Cytokine release syndrome	1	1	0	0	0	**2**
Cancer related						
Pain in tumor	8	9	1	0	0	**18**
Disease progression	0	0	1	0	4	**5**
Miscellaneous						
COVID-19	2	1	0	0	0	**3**
Non-specified infection	0	1	2	0	0	**3**
Hyponatremia	1	1	0	0	0	**2**
Total	**110**	**69**	**19**	**3**	**9**	**210**
Total per patient	**5.5**	**3.5**	**1.0**	**0.2**	**0.5**	**10.5**

Supplementary Table 4.

Cancer type(s)/subtype(s)/stage(s)/condition	Advanced solid cancers
Considerations related to:	
Sex	Of 18.1. million cancer cases worldwide, 9.3 million (51%) cases were male and 8.8 million (49%) cases were female [1].
Age	The most common age of diagnosis for cancer of any site is 66 years in the US [2].
Race/ethnicity	White or Caucasian patients account for the highest rates for new diagnosed cancers, whereas Black or African American patients account for the highest cancer mortality in the US [3].
Geography	Cancer accounted for nearly 10 million deaths worldwide in 2020 [4].
Overall representativeness of this study	This study enrolled 20 patients, of which 13 (65%) were female and 7 (35%) were male. Thus the study had a higher proportion of females than the average cancer population.<br><br>The median age of the participants was 58 years, less than the average cancer population. Ethnicity data was no collected in the present study, but due to the limited number of locations included in the trial, the study cannot represent the global cancer population.<br><br>Similarly, the study was geographically located in two centers in Helsinki, Finland and thus does not represent geographical differences in cancer care encountered across the world.

Supplementary Table 5. Representativeness of Study Participants.

[1] https://www.wcrf.org/cancer-trends/worldwide-cancer-data, accessed 10th December 2023.
[2] https://www.cancer.gov/about-cancer/causes-prevention/risk/age, accessed 10th December 2023.
[3] https://seer.cancer.gov/statfacts/html/disparities.html, accessed 10th December 2023.
[4] https://gco.iarc.fr/today, accessed 10th December 2023

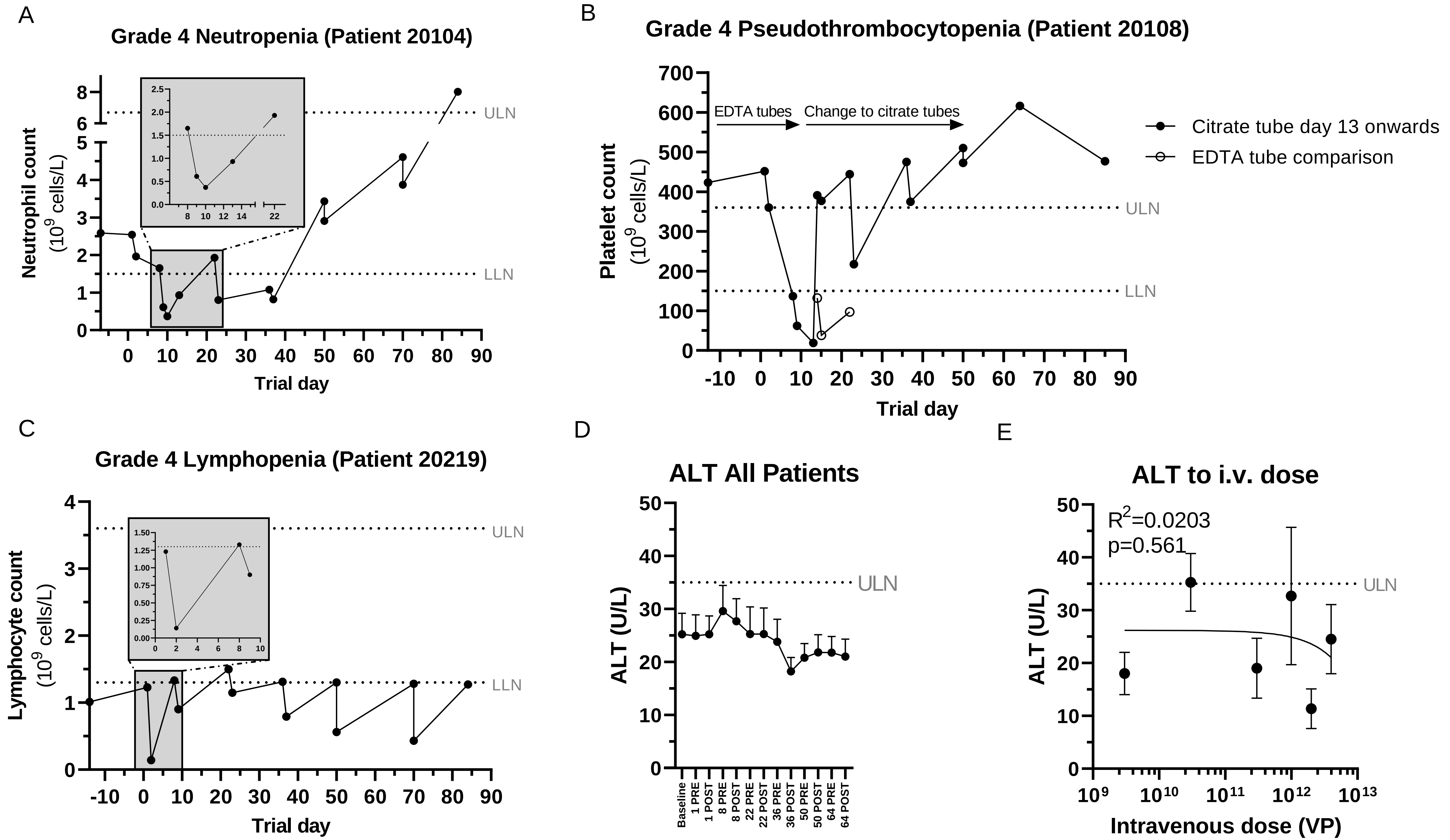

Supplementary Figure 1.

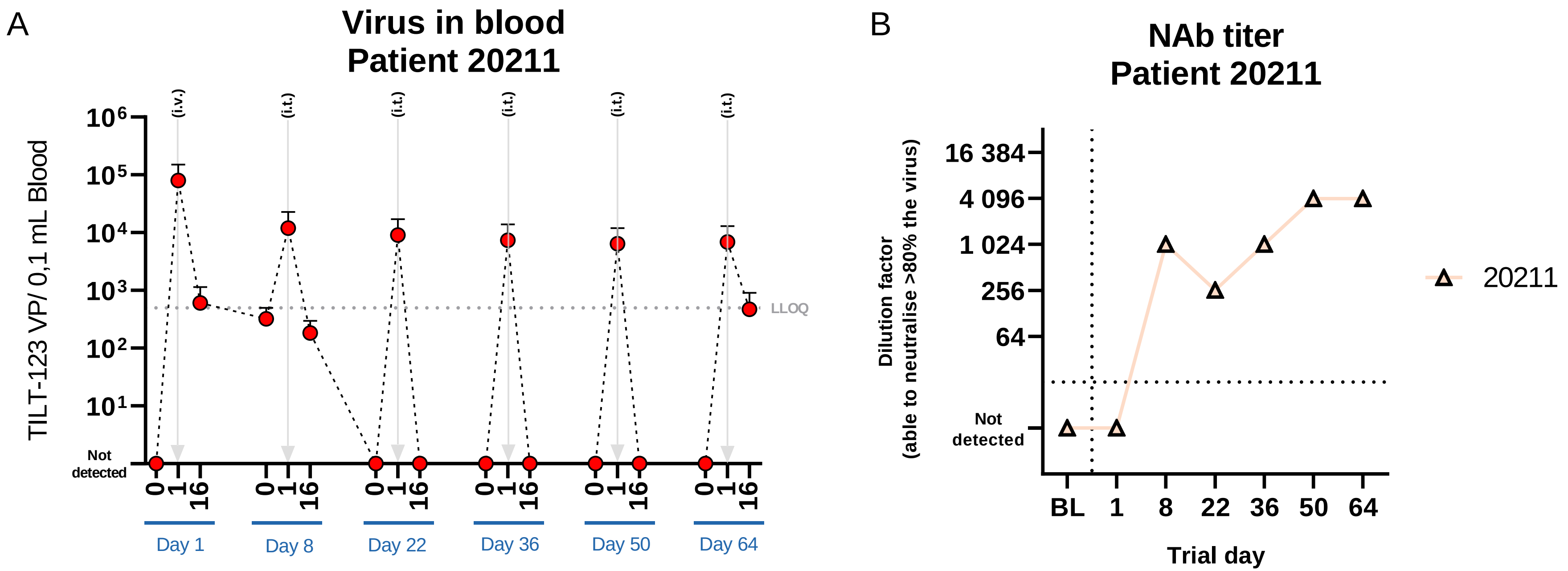

Supplementary Figure 2.

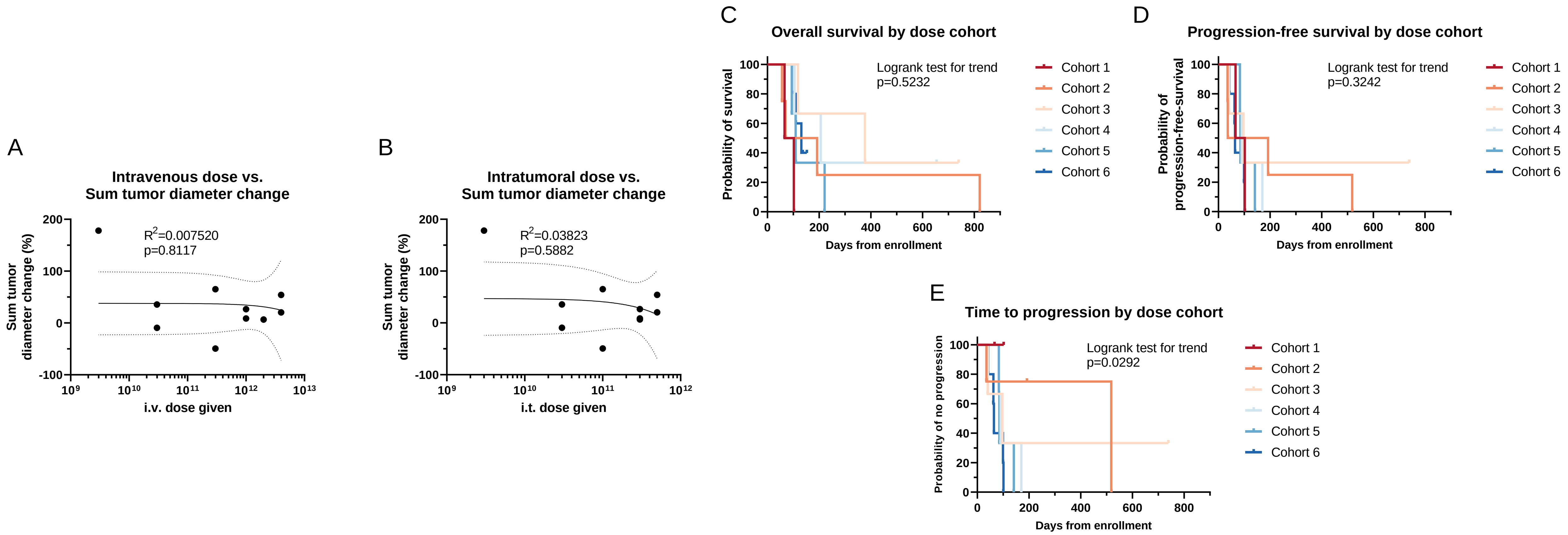

Supplementary Figure 3.

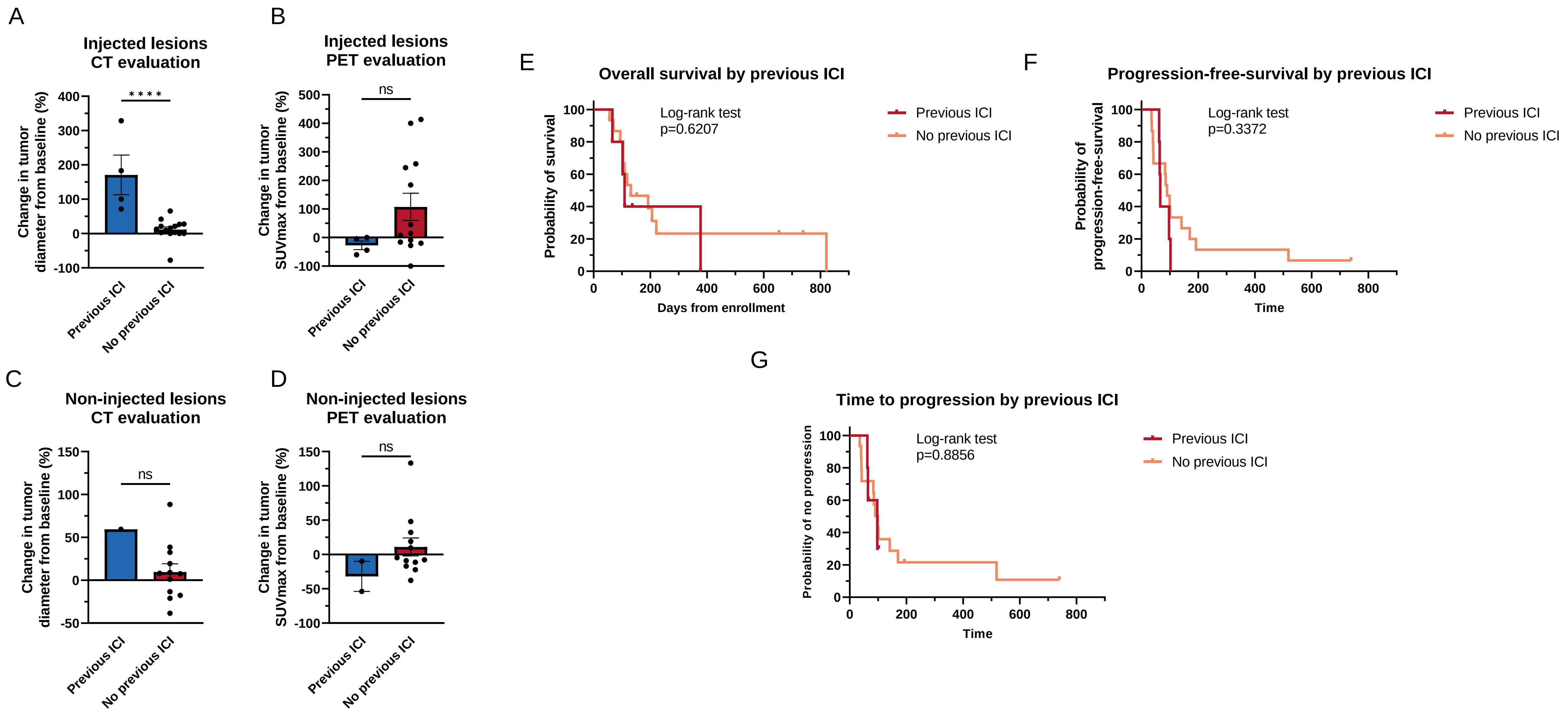

Supplementary Figure 4.

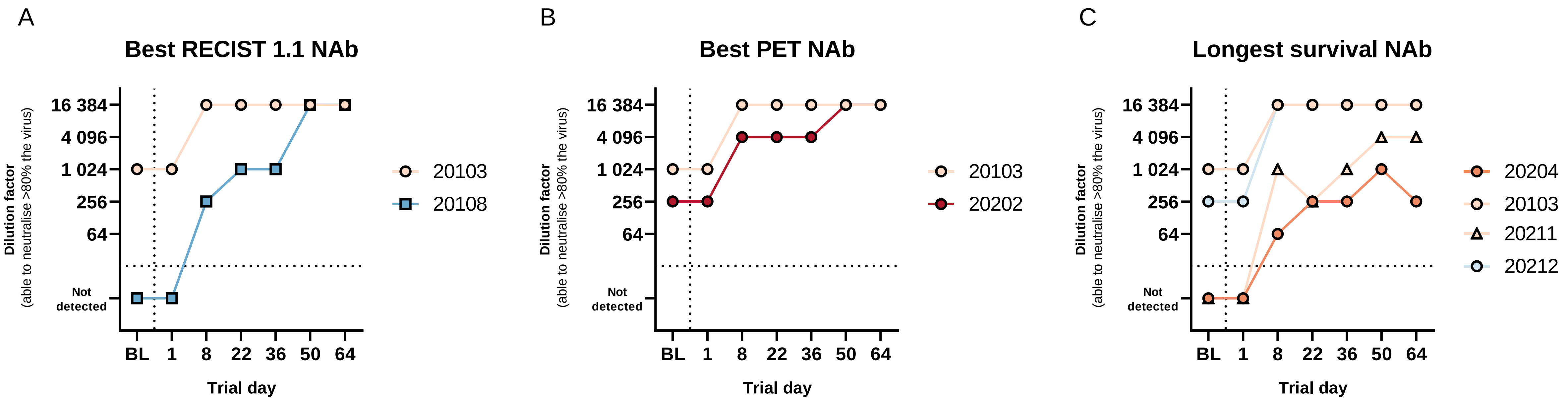

Supplementary Figure 5.

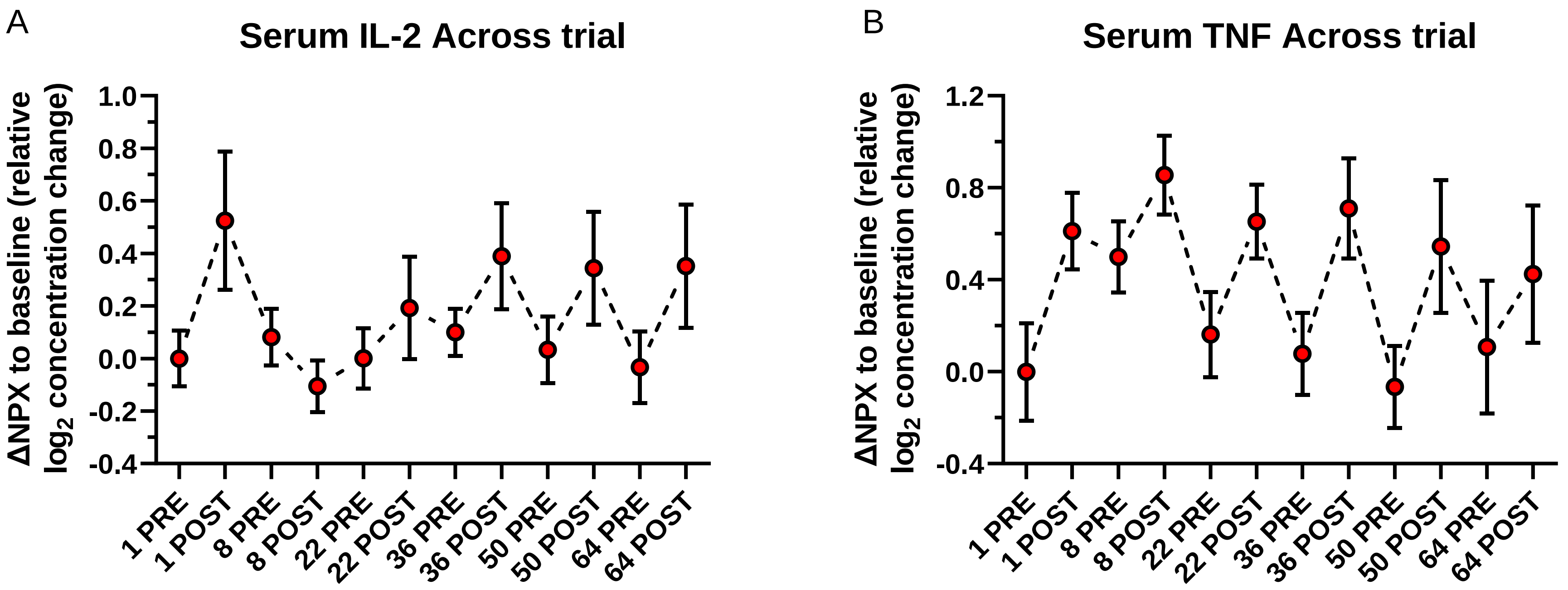

Supplementary Figure 6.

Supplementary Table 1. Antibodies used for adenoviral E1A staining and immunohistochemistry analysis.

Supplementary Table 2. PET criteria used in the study, based on Koski et al. [22].

Supplementary Table 3. Full patient characteristics, responses at the end of the trial or extension and progression-free survival. If patient continued to extension, best response shown. Data cut-off 26 Nov 2023. For progression-free survival and time to progression, patient was classified by best response across RECIST/iRECIST/PET criteria at day 78. For assessments not on day 78 or in extension protocol, physician evaluation of progression or any available imaging classified patient as progressing. N/A = not available, patient left the trial before imaging. PR = partial response. SD = stable disease. PD = progressive disease. CMR = complete metabolic response. PMR = partial metabolic response. SMD = stable metabolic disease. PMD = progressive metabolic disease.

Supplementary Table 4. All adverse events reported in the trial with >1 occurrence, related or unrelated to TILT-123 therapy. Events reported as of 11 November 2023.

Supplementary Table 5. Representativeness of Study Participants.

Supplementary Figure 1. A. Grade 4 neutropenia encountered in one patient (20104) on dose level 4/6, with resolution of neutropenia later in the trial. B. Grade 4 pseudothrombocytopenia encountered in one patient (20108) on dose level 5/6, with comparisons between EDTA and citrate tubes shown for days 14, 15 and 22, and resolution of low thrombocyte counts after change to citrate tubes C. Grade 4 lymphopenia encountered in one patient (20219) on dose level 6/6 after intravenous dose of TILT-123, with normalization and subsequent decreases in lymphocyte counts after each dose. D. Liver toxicity measured by alanine aminotransferase (ALT) across trial, all patients pooled. Mean +/- SEM shown. E. ALT level 24-48 hours after intravenous stratified by intravenous dose given and linear regression fit. Mean +/- SEM shown. LLN = lower limit of normal. ULN = upper limit of normal.

Supplementary Figure 2. A. Viral particles detected in the blood by qPCR for patient 20211. B. Neutralizing antibody titer for patient 20211.

Supplementary Figure 3. A. Intravenous dose given versus sum tumor diameter change on day 78. B. Intratumoral dose given versus sum tumor diameter change on day 78. For A-B, linear fit shown with 95% confidence intervals shown with R-squared for Goodness of Fit and p-value for slope deviation from zero. C. Overall survival by dose cohort. D. Progression-free survival by dose cohort. E. Time to progression by dose cohort. For E-G, trend measured with Logrank test for trend.

Supplementary Figure 4. Comparison of treatment effect by previous immune checkpoint inhibitor (ICI) therapy. A. Injected lesion diameter change evaluated by CT. B. Injected lesion SUVmax change evaluated by PET. C. Non-injected lesion diameter change evaluated by CT. D. Non-injected lesion SUVmax change evaluated by CT. For A-D, groups compared with two-sided t-test. E. Overall survival in the trial stratified by previous ICI therapy. F. Progression-free survival in the trial stratified by previous ICI therapy. G. Time to progression in the trial stratified by previous ICI therapy. For E-G, groups compared with Mantel-Cox Logrank test. **** = $p<0.001$. ns = non-significant ($p > 0.05$).

Supplementary Figure 5. A. Neutralizing antibodies across trial in patients with best RECIST1.1 responses. B. Neutralizing antibodies across trial in patients with best PET criteria responses. C. Neutralizing antibodies across trial in patients with longest survival.

Supplementary Figure 6. A. Changes in serum interleukin-2 across all trial treatment timepoints. B. Changes in serum tumor necrosis factor alpha across all trial treatment timepoints. Mean +/- SEM shown.
